# Inactivation of HCV and HIV by microwave: a novel approach for prevention of virus transmission among people who inject drugs

**DOI:** 10.1038/srep36619

**Published:** 2016-11-18

**Authors:** Anindya Siddharta, Stephanie Pfaender, Angelina Malassa, Juliane Doerrbecker, Anggakusuma, Michael Engelmann, Boya Nugraha, Joerg Steinmann, Daniel Todt, Florian W. R. Vondran, Pedro Mateu-Gelabert, Christine Goffinet, Eike Steinmann

**Affiliations:** 1 Institute of Experimental Virology, Twincore, Centre for Experimental and Clinical Infection Research; a joint venture between the Hannover Medical School (MHH) and the Helmholtz Centre for Infection Research (HZI) , Hannover, Germany; 2 Department of Rehabilitation Medicine, Hannover Medical School, Hannover, Germany; 3 Institute of Medical Microbiology, University Hospital Essen, Essen, Germany; 4 ReMediES, Department of General, Visceral and Transplantation Surgery, Hannover Medical School, and German Centre for Infection Research, Hannover-Braunschweig, Germany; 5 National Development Research Institutes, New York, United States

## Abstract

Hepatitis C virus (HCV) and human immunodeficiency virus (HIV-1) transmissions among people who inject drugs (PWID) continue to pose a challenging global health problem. Here, we aimed to analyse a universally applicable inactivation procedure, namely microwave irradiation, as a safe and effective method to reduce the risk of viral transmission. The exposure of HCV from different genotypes to microwave irradiation resulted in a significant reduction of viral infectivity. Furthermore, microwave irradiation reduced viral infectivity of HIV-1 and of HCV/HIV-1 suspensions indicating that this inactivation may be effective at preventing co-infections. To translate microwave irradiation as prevention method to used drug preparation equipment, we could further show that HCV as well as HIV-1 infectivity could be abrogated in syringes and filters. This study demonstrates the power of microwave irradiation for the reduction of viral transmission and establishment of this safety strategy could help reduce the transmission of blood-borne viruses.

Hepatitis C virus (HCV) infections constitute a major health problem with an estimated 80 million people being chronically infected^1^. In industrialized countries, injecting drug use is the major risk factor of acquiring an infection with 50–80% of HCV infection occurring in people who inject drugs (PWID)^2^. Different patient isolates can be grouped into 7 genotypes which differ in respect to global prevalence as well as treatment susceptibility^3^,^4^. Especially the HCV genotypes 1a, 1b and 3a are common among PWID, with genotype 4d being most common among PWID in Europe^5^,^6^,^7^. Current standard therapy, which consists of a combination therapy of one or more direct acting antivirals (DAAs) with or without pegylated interferon and ribavirin, is highly efficient with cure rates of over 90%, reduced toxicity, and shortened treatment duration^8^. However, high costs and general barriers in the antiviral management of PWID, including lack of knowledge and infrastructure as well as insufficient awareness of the patients hamper successful treatment of viral infection^9^,^10^. Furthermore, as a prophylactic vaccine is still lacking, re-infection even after successful therapy is possible, which constitutes a problem especially in patient populations with continued exposure to HCV^11^. It is estimated that around 10 million HCV-infected PWID face a high risk to develop liver fibrosis, cirrhosis and hepatocellular carcinoma^9^,^12^. Factors facilitating disease progression include age, alcohol abuse, obesity, insulin resistance and co-infection with human immunodeficiency virus type 1 (HIV-1)^13^. Indeed, HCV/HIV-1 co-infections pose a serious problem, owing to the shared modes of transmission with 20–30% of HIV-1-infected individuals being co-infected with HCV, which results in a greater risk of progression to liver disease as well as acceleration of the clinical course of HIV-1 infection^14^. Transmission of both pathogens occurs by sharing of contaminated needles and equipment (including syringes, water and filters)^15^,^16^. Prevention strategies including needle and syringe exchange programs and opioid substitution therapy, which have been successfully implemented to reduce HIV-1 incidences among PWID, but have been less effective for HCV prevention, probably due to a high prevalence among PWID as well as a 10 times higher infectivity of HCV compared to HIV-1^9^. Nowadays, deaths related to HCV even surpass the number of AIDS-related deaths^17^,^18^ and globally it is expected that the burden of HCV infection will even more increase within the next few decades^19^. To reduce further transmission of HCV and HIV-1 among PWID a general awareness about potential risk factors is necessary and feasible solution-orientated standard procedures should be established to lower the global burden of viral infection. In this study, we explored the effect of microwave irradiation on the stability and inactivation of HCV as well as HIV-1 as a simple method to prevent viral infections among PWID.

## Results

### Microwave irradiation reduces infectivity of all HCV genotypes

**Figure 1:**
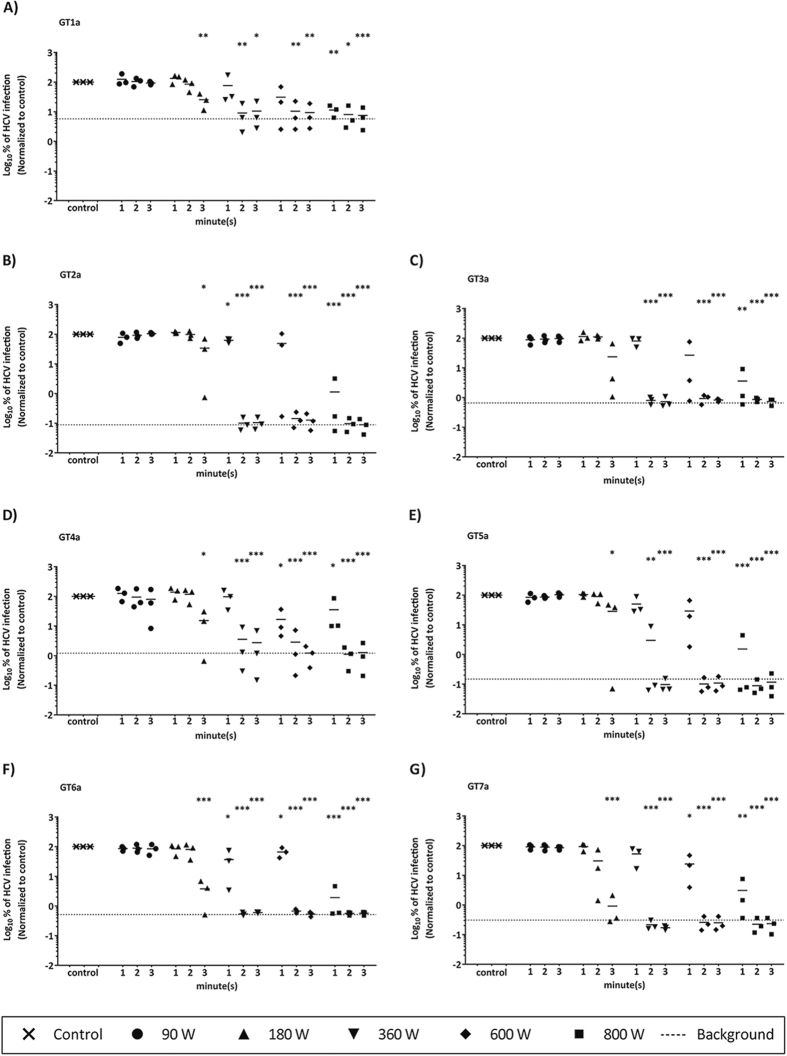
Infectivity of all HCV genotypes (GT 1–7) after microwave irradiation. Different HCV reporter virus genotypes ((**A**) GT1a, (**B**) GT2a, (**C**) GT3a, (**D**) GT4a, (**E**) GT5A, (**F**) GT6a, (**G**) GT7a) were exposed to microwave irradiation for different time durations and power levels. Viral infectivity was determined by luciferase reporter activity. Data were normalized to non-treated virus control. Dashed line represents the assay background. Depicted in Fig. 1A–G are data of three individual experiments (*p < 0.05, **p < 0.01, ***p < 0.001).

Microwave ovens are popular tools primarily used as quick food heating devices. They use electro-magnetic energy to rapidly heat dielectric materials, such as water^20^. We have previously analyzed the stability of HCV in liquids^21^ as well as on dried surfaces^22^ and reported HCV temperature sensitivity in a drug transmission assay^22^. To translate these findings into a preventive strategy for blocking virus transmission among PWID, microwave irradiation as a potential heating inactivation procedure against blood-borne viruses was investigated. HCV containing cell culture fluids (100 μL) were exposed to microwave irradiation for 1, 2 or 3 min at 90, 180, 360, 600 or 800 W (watt) and the viral titers of all seven different HCV genotypes were monitored (Fig. 1). Low power irradiation at 90 or 180 W had no influence on viral infectivity independent of the viral genotype (Fig. 1A–G). However, viral infectivity of all tested HCV genotypes could be already significantly reduced upon irradiation for at least 2 min at 360 W or higher power. Of note, shorter irradiation periods, even with higher powers, e.g. irradiation for 1 min at 600 or even 800 W, did not always result in a significant loss of viral infectivity as the time to reach a critical temperature was too short. As expected, an increase in irradiation time as well as power resulted in an increase in temperature, which was monitored for every treated culture fluid (Table 1). For example, under conditions of 180 W for 2 min, temperatures of about 42–53 °C were reached in the different culture fluids, whereas under conditions of 360 W 58–69 °C was recorded after 2 min of irradiation (Table 1). To directly compare the microwave irradiation sensitivity between different HCV genotypes, the temperature which is necessary to reduce the infectivity for each HCV genotype by 90% (inhibitory temperature 90: IT_90_) was calculated from the data points. As depicted in Suppl. Fig. 1, genotype 3 viruses were most sensitive to microwave irradiation with an IT_90_ of 52 °C, whereby the other HCV genotypes IT_90_ values ranged between 56–60 °C indicating that this decontamination practice is equally effective against all HCV genotypes (Suppl. Fig. 1).

**Figure 2:**
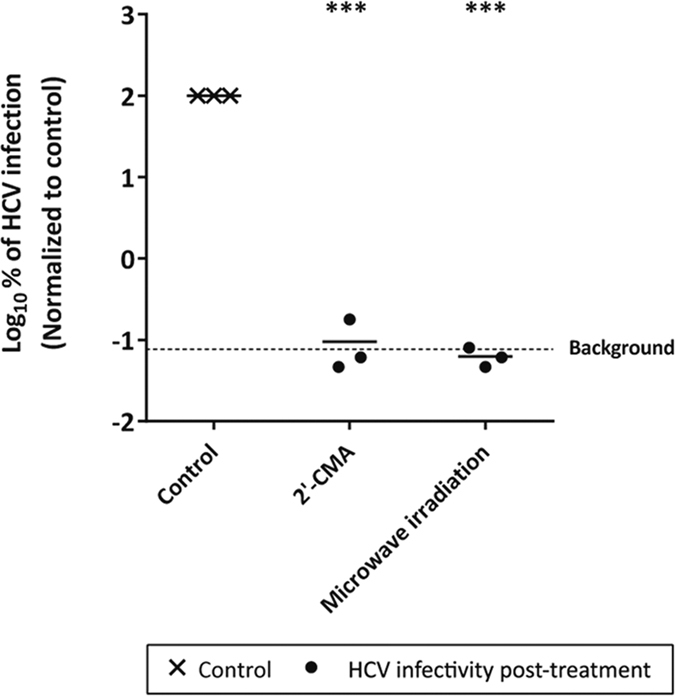
HCV infection of PHH after microwave irradiation. Supernatant containing HCV (Jc1) was irradiated in the microwave oven for 2 min with 800 W and then used to infect PHH. *De novo* production of infectious virus after 24 h was monitored by determination of the tissue culture infection dose (TCID_50_/mL). As a positive control, an HCV polymerase inhibitor (2′-CMA) (end-concentration 1 μM 2′-CMA) was used. Data were normalized to non-treated virus control. Dashed line represents the assay background. Depicted are data of three individual experiments (***p < 0.001).

Importantly, the effect of microwave inactivation procedure could be confirmed with a high viral titer stock of non-reporter Jc1 wildtype virus (Suppl. Fig. 2) and upon infection of *ex vivo* isolated primary human hepatocytes (PHH) that were infected with HCV cell culture fluids. *De novo* production of infectious virus could be significantly reduced in PHH by treatment of HCV with 800 W for 2 min, while an HCV polymerase inhibitor (2′-C-methyladenosine; 2′-CMA) was used as control (Fig. 2). In summary, infectivity of HCV from all genotypes in human liver cells could be efficiently abrogated after microwave irradiation with 600 W for at least 2–3 min.

### Infectivity of HCV/HIV-1 co-infection is reduced to undetectable levels by microwave irradiation

**Figure 3:**
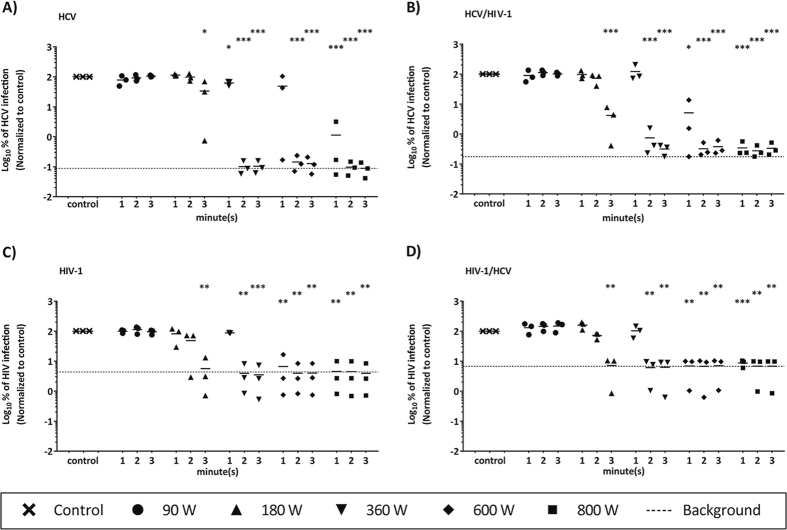
Stability of HCV and/or HIV-1 after microwave irradiation. Suspension of reporter virus HCV (Jc1) in a mono-incubation setup (**A**) and co-incubation with HIV-1 (**B**), as well as HIV-1 in a mono-incubation setup (**C**) and in co-incubation with HCV (**D**) were exposed to microwave irradiation and viral titers were determined for HCV and HIV-1 by measuring reporter luciferase activity. Data were normalized to non-treated virus control. Dashed line indicates assay background. Depicted are the results of three individual experiments (*p < 0.05, **p < 0.01, ***p < 0.001).

Due to similar modes of transmission, HCV/HIV-1 co-infection is very common especially among PWID, with one-third of HIV-1-infected Americans, and 7 million people worldwide being co-infected^23^,^24^. To analyze the effect of microwave irradiation upon HCV/HIV-1 co-contamination, HCV (genotype 2a Jc1 virus) or HIV-1 were either individually irradiated as described above or co-incubated to mimic a co-infection, before microwave irradiation was performed. As observed before, low irradiation power (90 or 180 W) did not significantly reduce individual HCV (Fig. 3A) or HIV-1 (Fig. 3C) single infectivity. However, both viruses could be significantly inactivated upon irradiation for at least 2 min and 360 W. This reduction in viral titers could consistently be observed in the co-incubation setup, with both HCV (Fig. 3B) as well as HIV-1 (Fig. 3D) irrespective of the presence of another virus. This indicates that microwave irradiation is effective against HCV as well as HIV-1 infectivity and could contribute to the prevention of virus transmission even in the context of co-exposure.

### Microwave irradiation of contaminated syringes abrogates HCV as well as HIV-1 infectivity

**Figure 4:**
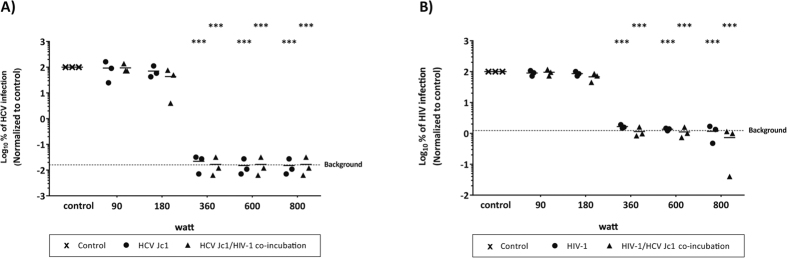
Stability of HCV and/or HIV-1 in syringes after microwave irradiation. Suspensions of wildtype HCV (Jc1) and/or HIV-1 were spiked into an insulin syringe and irradiated for 3 min at different powers. Virus suspension was collected from the syringe and viral titers were determined for HCV (**A**) by limiting dilution assay to determine the tissue culture infection dose (TCID_50_/mL) and for HIV-1 (**B**) by determining reporter luciferase activity. Data were normalized to the non-treated virus control. Dashed line indicates assay background. Depicted are the results of three individual experiments (***p < 0.001).

Sharing of contaminated drug preparation and injection equipment, including syringes and filters, among PWID is thought to be the main route for viral transmission^15^. To mimic such a transmission scenario, syringes were contaminated with infectious HCV and/or HIV-1 viral particles and irradiated for 3 min with different microwave powers. HCV infectivity within syringes was significantly reduced upon irradiation at 360 W or higher, with no differences between single HCV infection or HCV/HIV-1-co-infection (Fig. 4A). Accordingly, HIV-1 titers could be reduced to background levels upon irradiation at 360 W independent of HCV co-infection (Fig. 4B) demonstrating that microwave irradiation also reduces efficiently virus infectivity of contaminated syringes.

### Inactivation of HCV and/or HIV-1 in contaminated drug preparation filters after microwave irradiation

**Figure 5:**
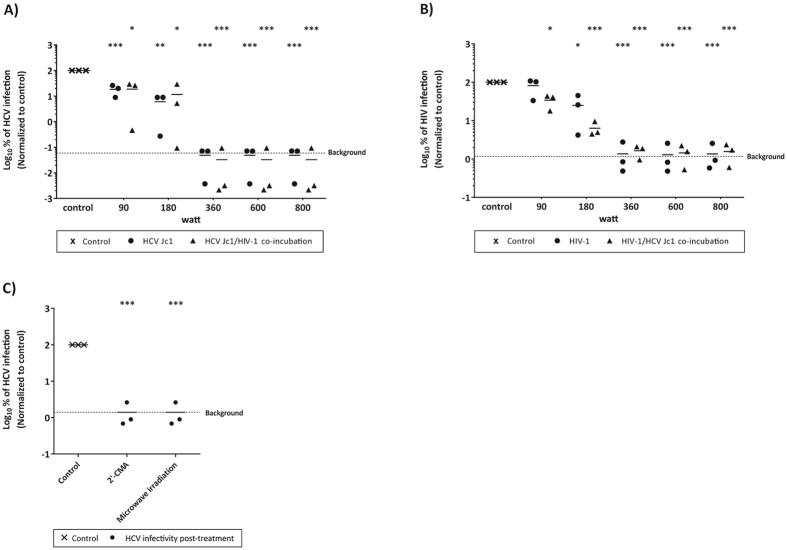
Stability of HCV and HIV-1 in cigarette filters after microwave irradiation. Slim cigarette filters were drenched in wildtype HCV (Jc1) and/or HIV-1 suspensions. Viral suspension was removed from the filter using an insulin syringe and the filter was irradiated for 3 min at different powers. Residual viral particles were recovered from the filter by adding 400 μL of cell culture medium and incubating it for 5 min at 37 °C under shaking. Insulin syringe was used to draw the supernatant from the filter. The viral titers were determined for HCV (**A**) by the tissue culture infection dose (TCID_50_/mL) and for HIV-1 (**B**) by determining reporter luciferase activity. Data were normalized to non-treated virus. Dashed line indicates assay background. For the experiment in PHH (**C**), the HCV contaminated filter was irradiated for 2 min with an irradiation power of 800 W. *De novo* production of infectious virus after 24 h was monitored by determination of the tissue culture infection dose (TCID_50_). Depicted are the results of three individual experiments (*p < 0.05, **p < 0.01, ***p < 0.001).

In addition to contaminated syringes, re-use of virus-positive drug preparation cigarette filters constitutes a possible source of infection^22^. Filters are commonly used by PWID to remove impurities from the drug solution and to prevent needle blockage on injection, but are also re-used due to residual amounts of drugs that resided within the filter. Infectious virus particles have been demonstrated to remain infectious in such drug preparation filters from several hours up to 2 days when wrapped in foil^22^ and therefore represents a high risk for virus cross-transmission among PWID upon sharing. To assess the antiviral effect of microwave irradiation on this drug paraphernalia, drug preparation filters were contaminated with HCV and/or HIV-1 as described before^22^ and the residual viral titer was determined after microwave irradiation for 3 min. Whereas low power irradiation (90 or 180 W) did not reduce viral infectivity, both HCV (Fig. 5A) as well as HIV-1 (Fig. 5B) infectivity, either upon single infection or in the co-incubation setup, could be significantly reduced after irradiation at 360 W or more (Fig. 5). These results were recapitulated in the context of HCV infection of PHH after HCV drug preparation filter contamination and microwave inactivation (Fig. 5C).

**Figure 6:**
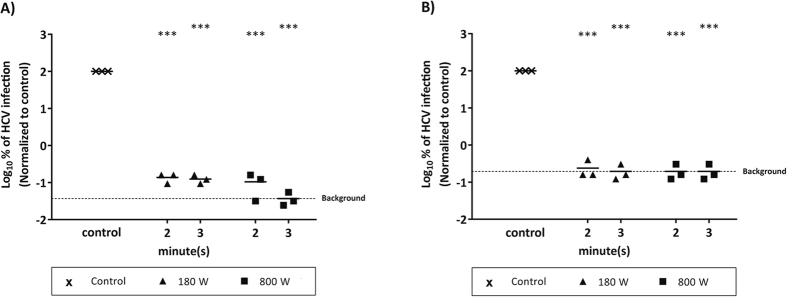
Microwave inactivation of HCV in air-dried filters. Slim cigarette filters were drenched in wildtype HCV (Jc1) suspensions. Viral suspension was removed from the filter using an insulin syringe. Afterwards the filter was wrapped in plastic foil and was left under the hood for either 6 h (**A**) or 24 h (**B**) following irradiation for 3 min at different powers. Residual viral particles were recovered from the filter by adding 400 μl of cell culture medium and incubation for 5 min at 37 °C under shaking. Insulin syringe was used to draw the supernatant from the filter. The viral titers were determined for HCV by limiting dilution assay to determine the tissue culture infection dose (TCID_50_/mL). Data were normalized to mock – non treated virus. Dashed line indicates assay background. Depicted are the results of three individual experiments (***p < 0.001).

**Figure 7:**
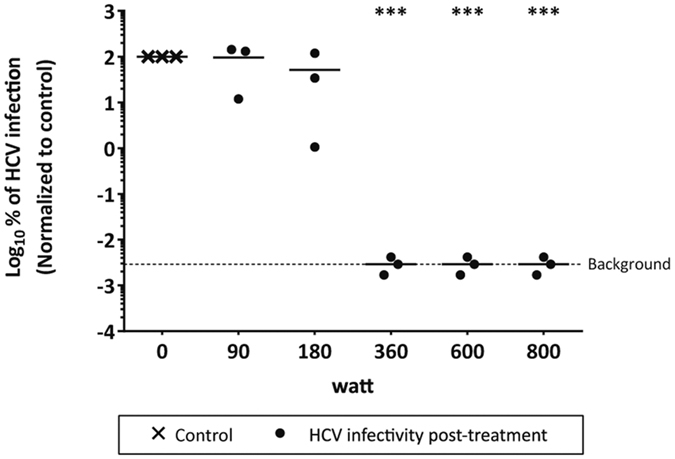
Microwave inactivation of HCV in filters in the presence of heroin. 1 mg/μL street heroin was spiked into wildtype HCV (Jc1) containing virus suspension. A slim cigarette filter was drenched in this solution. Viral suspension was removed from the filter using an insulin syringe and the filter was irradiated for 3 min at different powers. Residual viral particles were recovered from the filter by adding 400 μL of cell culture medium and incubating it for 5 min at 37 °C under shaking. Insulin syringes were used to draw the supernatant from the filter. The viral titers were determined for HCV by limiting dilution assay to determine the tissue culture infection dose (TCID_50_/mL). Data were normalized to non-treated virus control. Dashed line indicates assay background. Depicted are the results of three individual experiments (***p < 0.001).

To mimic also the potential storage of filters for hours or days by PWID, we next air-dried the drug preparation filters after viral contamination for either 6 h (Fig. 6A) or 24 h (Fig. 6B) and then applied the microwave inactivation procedure. Importantly, also air-dried filter associated virus could be significantly reduced upon irradiation by 180 or 800 W for 2 or 3 min (Fig. 6A,B). Finally, we simulated an even more physiological setup of virus-contaminated drug preparation equipment by adding 1 mg/μL of street heroin to an HCV suspension. We used this virus/drug solution to contaminate drug preparation filters as described and performed microwave irradiation of the filters in a dose-dependent manner for 3 min. Even in the presence of the drug, viral infectivity could be significantly reduced upon irradiation at 360 W or higher (Fig. 7). In conclusion, these results show that microwave irradiation of virus contaminated filters with drug preparations can efficiently inactivate viral infectivity and thereby reduces the transmission risks among PWID.

## Discussion

Viral transmission among PWID is a global public health problem with approximately 10 million PWID being infected with HCV and 3 million with HIV-1, including a high percentage of HCV/HIV-1 co-infected individuals^12^,^25^,^26^. Whereas harm reduction programs and opiate substitution treatments significantly decreased HIV-1 prevalence and incidence in recent years, HCV transmission continue to remain high with HCV incidences ranging from 5 to 45% per year^10^,^27^,^28^. New prevention strategies are necessary to help reducing the global transmission of HCV and HIV-1 especially among PWID. In this study, we aimed to analyze a universally applicable procedure, namely microwave irradiation, as a method to inactivate viral contaminations in syringes and filters and therefore to reduce potential viral transmission due to sharing infected equipment. Microwave ovens are commonly used as quick food heating devices. Previous studies have shown before that pasteurisation (heat treatment at 60 °C for several hours) can inactivate viral infectivity of HCV, HBV as well as HIV-1^29^. For HCV it has been demonstrated that heat administration in the range of 60 °C or higher is sufficient to destroy viral infectivity^30^ with mainly the viral envelope but also the viral RNA being affected by this procedure^31^. In the experimental conditions applied here, microwave irradiation for at least 2 min or longer at not less than 600 W is sufficient to reach a critical temperature which facilitates inactivation of HCV as well as HIV-1 or HCV/HIV-1. Viral transmission between PWID is often facilitated by sharing of contaminated drug preparation equipment^16^,^32^,^33^,^34^. Indeed, several studies reported that HCV is very resilient and capable of surviving on drug preparation equipment including needles, syringes, filter and water over a long period^22^,^30^,^35^. By mimicking the re-use of potentially contaminated syringes and filters, which are both commonly shared among PWID, we could show that microwave irradiation of this drug preparation equipment can effectively reduce viral infectivity; therefore preventing potential cross-transmission of viruses between individuals. This could be further observed even in the presence of street heroin. A limitation of the microwave-based inactivation of HCV in contaminated syringes is the metal-derived needle, which should not be placed in the microwave. Therefore, we recommend removing and disposing the needle or decontaminating the needle using disinfection reagents that will inactive HCV^21^,^22^. In case of syringes where the needle cannot be removed, the virus microwave inactivation procedure is not recommended. As UV- light was also reported to inactivate HCV at least in blood products^36^, it would be interesting to evaluate UV-light as a potential source for virus inactivation in future studies.

Importantly, the temperature required to inactivate virus particles should not impact the heroin drug effect as the major component of street heroin, diacetylmorphine, begins to melt at 173 °C^37^. This high melting temperature of heroin and usual practice of PWID to cook heroin and other drugs into solution further suggests that our microwave heating approach will not destroy the stability of heroin. At 800 W heating for 3 minutes a maximum temperature of 90 °C was achieved as indicated in Table 1, which is far below the melting point of heroin. Cocaine has a melting point of 98 °C and boiling point of 187 °C and is reported to be stable at 65 °C for up to one month^38^,^39^. The temperature required to reduce the infectivity of all HCV genotypes by 90% was determined between 56–60 °C, which could already be reached with a power of 360 W for 2 min. Future studies could deal with a fine-tuning of the microwave frequency to the virus resonance frequency as previously described for influenza viruses^40^ to reduce the heating effect and thereby improve the blood-borne virus inactivation method.

PWID represent the core of the HCV epidemic particularly in high- and middle-income countries and with no protective vaccine available transmission of HCV as well as HIV-1 continues^19^. Even though treatment options have increased substantially and treatment paradigms are changing, there are still several barriers which prevent successful treatment of PWID^9^,^41^. In situations where sterile drug injection equipment is not readily available, a simple and quasi-universally applicable procedure such as microwave heating for a limited time could be promoted to reduce viral transmission among PWID. We could show that by simply heating drug preparation equipment via microwave irradiation, viral infectivity of HCV and HIV-1 could be efficiently reduced. Promotion of this procedure should help to reduce and/or prevent transmission of blood borne viruses, like HCV and HIV-1, and facilitate a reduction in the global burden.

## Materials and Methods

### Plasmid and Viruses

The HCV plasmid pFK-Jc1 encodes for the intragenotypic 2a/2a chimeric virus Jc1^42^. Reporter HCV genotypes 1–7 (GT1a isolate H77, GT2a isolate Jc1, GT3a isolate S52, GT4a isolate ED43, GT5a isolate SA13, GT6a isolate HK6a, and GT7a isolate QC69) encompass intergenotypic chimeras encoding core - NS2 of genotype 1–7 and untranslated regions (UTRs) and NS3–NS5B of JFH1 and have been described before^43^,^44^,^45^. The plasmid encoding the full-length HIV-1 clone NL4.3 was used to generate HIV-1 particles as previously described^46^.

### Cell Culture

Huh7.5, HEK293T and TZM cells were cultured in Dulbecco’s modified Eagle’s medium (DMEM, Invitrogen) supplemented with 10% foetal bovine serum, 1% nonessential amino acids (Invitrogen), 100 μg/mL streptomycin (Invitrogen) and 100 IU/mL penicillin (Invitrogen). Primary human hepatocytes (PHH) were isolated from liver specimens obtained after partial hepatectomy, plated at a density of 1.3 × 10^6^ on collagen in P6 dishes, and kept in hepatocyte culture medium (Lonza) as described^47^.

### *In Vitro* Transcription, Electroporation, and Production of Cell Culture-derived HCV

Infectious HCV particles were produced as described previously^48^. Briefly, HCV plasmid DNA was linearized and transcribed into RNA, followed by the electroporation into Huh7.5 cells. Virus-containing culture fluids were harvested after 48 and 72 h, filtered through a filter with a pore size of 0.45 μm (VWR International) and used directly or further concentrated using centricons (Centricon Plus-70; Millipore, USA). For the determination of viral infectivity, cell-free supernatants were used to infect naive Huh7.5 target cells.

### Production of infectious cell culture derived HIV-1

For production of infectious HIV-1, HEK293T cells were transfected with a plasmid encoding full-length HIV-1_NL4.3_ by calcium-phosphate DNA precipitation (Takara Bio Europe). Virus-containing culture supernatants were harvested 48–72 h post transfection, filtered using 0.45 μm filters (VWR International), and concentrated via ultracentrifugation through a 20% sucrose cushion (Sigma-Aldrich). The infectious titer of the virus stock was defined by a β-galactosidase-based blue cell assay using TZM-bl cells^46^.

### Determination of HCV and HIV-1 Infectivity

Titers of infectious wildtype HCV virus (Jc1) were determined via limiting dilution assay on Huh7.5 cells as described elsewhere, with slight modifications^49^. The 50% tissue culture infectious dose (TCID_50_) was ascertained at 72 h post infection as described previously^42^. Luciferase reporter virus activity of the HCV genotypes was determined upon infection of Huh7.5 cells as described before^50^. Briefly, cells were lysed in luciferase lysis buffer (Promega) 72 h post infection. Renilla luciferase activity was measured after addition of the luciferase substrate (1 μmol/L coelenterazine; P.J.K) using a luminometer (Lumat LB9507).

HIV-1 infectivity was determined by a firefly luciferase-based blue cell assay using TZM-bl cells^46^. Infected cells were lysed in luciferase lysis buffer (Promega) according to the manufacturer’s instructions. Luciferase reporter activity was determined luminometrically after addition of luciferase substrate (Promega).

### Irradiation of HCV and/or HIV-1 by microwave

The microwave irradiation was performed in a household Bosch microwave oven type, serial number: MM817ASM (220–230 V, 2450 MHz) with a maximum power of 800 W. To investigate the efficacy of microwave irradiation against all HCV genotypes, 100 μL of cell-free infectious viral supernatant contained in an eppendorf tube was placed on a fixed position in the middle of the microwave, where the highest heat development occurs and irradiated for 1, 2, or 3 min with 90, 180, 360, 600 or 800 W. In the co-incubation setup, 50 μL of HCV containing culture fluid were mixed with HIV-1 (v/v) and the respective mono-incubations were performed in 50 μL of reporter HCV or HIV-1-containing supernatant which was diluted in 50 μL DMEM. *Ex vivo* experiments were performed by the infection of primary human hepatocytes (PHH) with HCV (Jc1). Aliquots of cell culture-derived HCV supernatants (100 μL) were irradiated in the microwave for 2 min with 800 W. As a positive control, an HCV polymerase inhibitor (2′-CMA) (end-concentration 1 μM 2′-CMA) was used. Microwave or 2′-CMA treated viral supernatants were used to infect PHH. After 6 h incubation at 37 °C, a medium change was performed by addition of hepatocyte culture medium (Lonza) as described^47^. *De novo* production of infectious virus after 24 h was monitored by TCID_50_.

### Microwave irradiation of contaminated syringes with HCV and/or HIV-1

Suspensions of HCV and/or HIV-1 were spiked into an insulin syringe (U-40 Insulin-1 mL/40 I.U. Omnifix 40 Solo; B. Braun Melsungen AG, Melsungen, Germany) without needle attachment. In the mono-incubation setup, 50 μL of reporter HCV (Jc1) or HIV-1 supernatant was diluted in 50 μL DMEM. In the co-incubation setup, 50 μL of HCV containing culture fluid were mixed with HIV-1 (v/v). The syringe together with the virus suspension was irradiated at different powers for 3 min in a microwave oven. The virus suspension was recovered from the syringe and the viral titers were determined as described above.

### Microwave irradiation of contaminated drug preparation cigarette filters with HCV and/or HIV-1

Slim cigarette filters, 6 mm × 15 mm (Gizeh, Gummersbach, Germany), were soaked in reporter HCV (Jc1) or HIV-1 containing culture fluids on the spoon with a volume of 200 μL diluted in 200 μL DMEM. In case of HCV/HIV-1 co-incubation settings, 200 μL of HCV containing culture fluid were mixed with HIV-1 (v/v). For some experiments, 1 mg/μL street heroin (40% 3,6-Diacetylmorphine, 8% 6-Acetylmorphine, 8% 6-Acetylcodeine, 12% Caffeine, 4% Codein, 4% Morphine, 8% Narcotine, 4% Papaverine, and 12% Paracetamol; LipoMed AG) with permission of the Federal Institute for Drugs and Medical Devices (BtM number 4606432) was added to 400 μL HCV supernatant with a viral titer of about 10^6^ TCID_50_/mL and slim cigarette filters were soaked within the mixture. Excess viral fluid was removed using an insulin syringe and contaminated filters were exposed to microwave irradiation at different powers for 3 min. To investigate the viral infectivity of HCV in air-dried cigarette filters, slim cigarette filters were soaked in HCV (Jc1) containing culture fluid with a volume of 400 μL and a viral titer of about 10^6^ TCID_50_/mL. Excess viral fluid was removed using an insulin syringe before the contaminated filters were placed under the fume hood for 6 or 24 h at room temperature, covered with standard household foil (Edeka, Hamburg, Germany). After indicated drying periods, contaminated filters were exposed to microwave irradiation at different powers for 3 min. Viral particles were recovered upon shaking of the filter in 400 μL DMEM for 5 min at 37 °C. Viral titers were determined as described above. Additionally, e*x vivo* experiments were also performed by the infection of PHH with HCV (Jc1) after contamination of slim cigarette filters and viral titers were determined as described above.

### Statistical Analyses

All experiments had an untreated virus control (100% of infection) and treatment group, distinguished by irradiation power 90, 180, 360, 600 or 800 W for either 1, 2, or 3 min. Each experimental treatment was performed in three biological replicates. Significant differences between control and each sample were determined using student’s *t*-test^51^. All data on each figure are presented on a logarithmic scale. Significance of the results was set as *p* < 0.05. Statistic package SPSS 23.0 (IBM Corp., Armonk, New York) was used for this evaluation. (*for p < 0.05, **for p < 0.01, ***for p < 0.001).

## Additional Information

**How to cite this article**: Siddharta, A. *et al*. Inactivation of HCV and HIV by microwave: a novel approach for prevention of virus transmission among people who inject drugs. *Sci. Rep.* **6**, 36619; doi: 10.1038/srep36619 (2016).

**Publisher’s note**: Springer Nature remains neutral with regard to jurisdictional claims in published maps and institutional affiliations.

## Supplementary Material

Supplementary Information

## Figures and Tables

**Table 1:** Temperature measurements for each HCV genotype sample after microwave irradiation.

Power (W)	Exposure Time (minute(s))	Temperature (°C)						
		GT1a	GT2a	GT3a	GT4a	GT5a	GT6a	GT7a
90	1	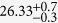	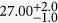	27.00	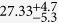	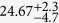	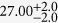	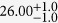
	2	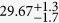	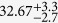	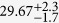	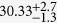	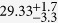	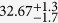	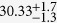
	3	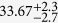	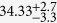	34.00	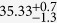	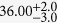	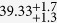	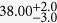
180	1	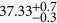	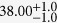	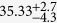	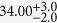	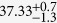	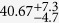	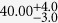
	2	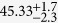	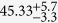	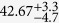	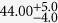	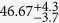	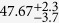	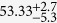
	3	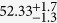	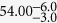	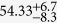	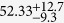	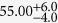	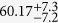	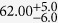
360	1	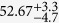	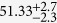	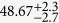	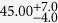	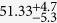	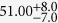	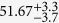
	2	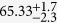	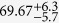	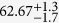	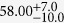	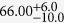	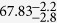	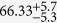
	3	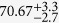	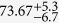	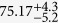	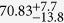	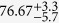	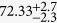	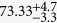
600	1	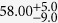	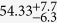	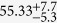	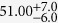	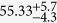	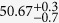	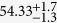
	2	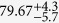	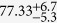	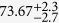	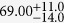	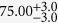	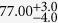	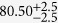
	3	87.00	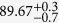	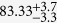	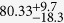	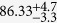	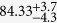	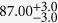
800	1	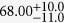	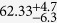	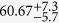	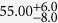	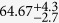	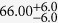	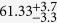
	2	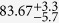	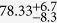	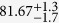	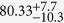	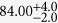	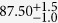	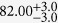
	3	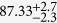	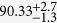	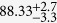	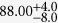	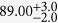	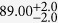	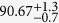
