# Supplementary material for: Inactivation of HCV and HIV by microwave: a novel approach for prevention of virus transmission among people who inject drugs
Source: Sci Rep. 2016 Nov 18;6:36619. doi: 10.1038/srep36619 (PMC5114683; doi:10.1038/srep36619)
Supplement: Supplementary Information [file srep36619-s1.pdf]

Supplementary information to:

Inactivation of HCV and HIV by microwave: a novel approach for prevention of virus transmission among people who inject drugs

Anindya Siddharta[1], Stephanie Pfaender[1], Angelina Malassa[1], Juliane Doerrbecker[1], Anggakusuma[1], Michael Engelmann[1], Boya Nugraha[2], Joerg Steinmann[3], Daniel Todt[1], Florian W.R. Vondran[4], Pedro Mateu-Gelabert[5], Christine Goffinet[1], and Eike Steinmann[1*]

[1]Institute of Experimental Virology, Twincore, Centre for Experimental and Clinical Infection Research; a joint venture between the Medical School Hannover (MHH) and the Helmholtz Centre for Infection Research (HZI)

[2]Department of Rehabilitation Medicine, Hannover Medical School, Hannover, Germany

[3]Institute of Medical Microbiology, University Hospital Essen, Essen, Germany

[4]ReMediES, Department of General, Visceral and Transplantation Surgery, Hannover Medical School, and German Centre for Infection Research, Hannover-Braunschweig

[5]National Development Research Institutes, New York, United States

Supplementary Figure 1

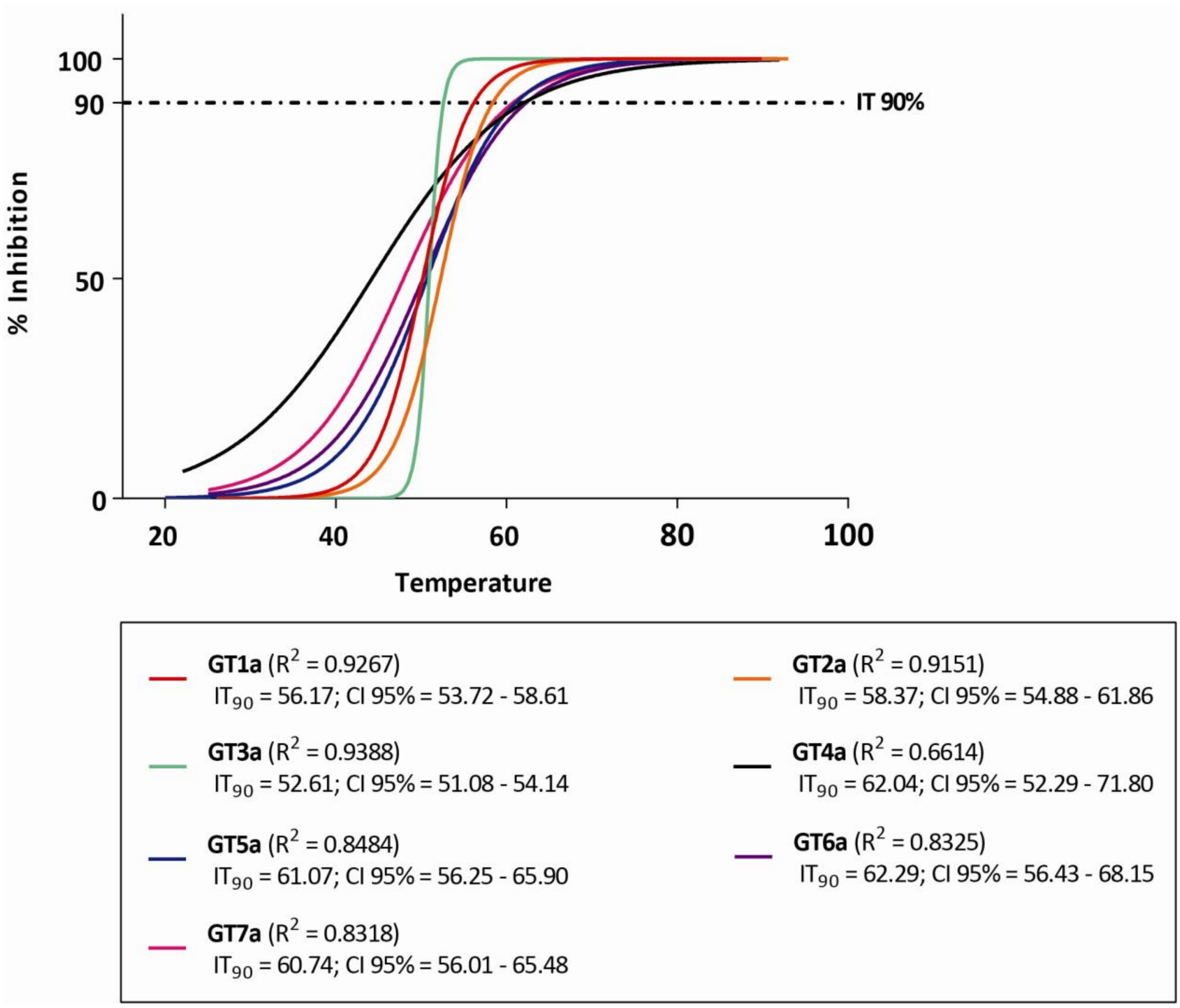

Determination of the inhibitory temperature 90 (IT_{90}) for all HCV genotypes. Dose-response curves of percent inhibition of viral infectivity (y-axis) against temperatures of virus supernatant after microwave treatment (x-axis) for all HCV genotypes are depicted. The effective temperature to reduce infectivity by 90% (inhibitory temperature 90%: IT_{90}; dot-dashed line) was calculated from three independent experiments using GraphPad Prism version 6.07 for Windows (GraphPad Software, La Jolla California USA, www.graphpad.com) and are inscribed with the respective 95% confidence intervals (CI) and coefficient of determination (R^2) in the box.

Supplementary Figure 2

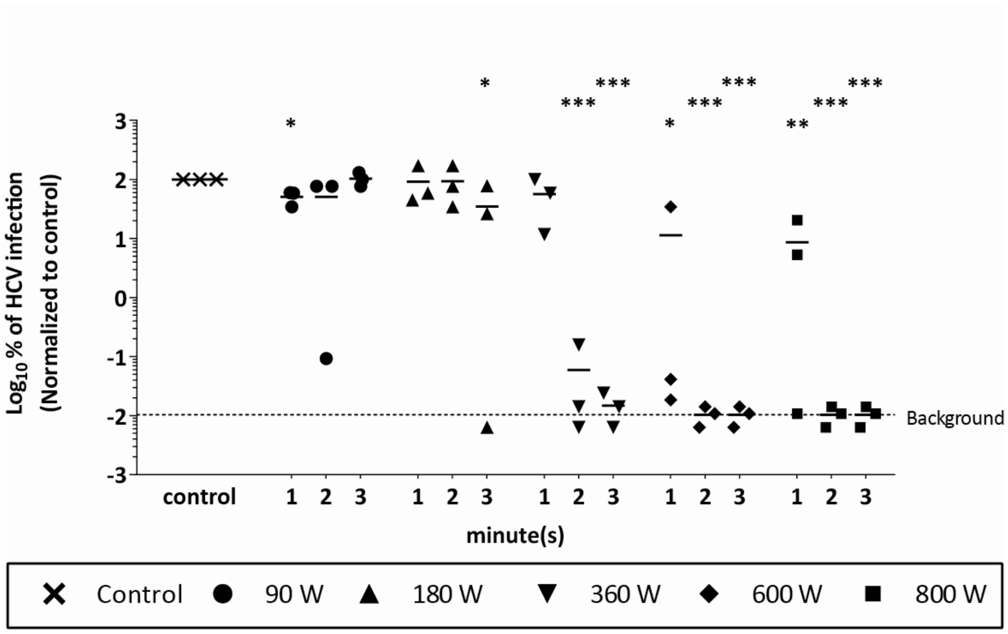

Stability of wildtype HCV (Jc1) after microwave irradiation. HCV wild type (Jc1) was exposed to microwave irradiation for different time durations and power levels and viral titers were determined by the tissue culture infection dose ($TCID_{50}$/mL). Data were normalized to non-treated virus controls. Dashed line represents assay background. Depicted are data of three individual experiments (*$p < 0.05$, **$p < 0.01$, ***$p < 0.001$).
